# Parents Accidentally Substitute Similar Sounding Sibling Names More Often than Dissimilar Names

**DOI:** 10.1371/journal.pone.0084444

**Published:** 2013-12-31

**Authors:** Zenzi M. Griffin, Thomas Wangerman

**Affiliations:** 1 Department of Psychology, University of Texas at Austin, Austin, Texas, United States of America; 2 School of Psychology, Georgia Institute of Technology, Atlanta, Georgia, United States of America; University of Leicester, United Kingdom

## Abstract

When parents select similar sounding names for their children, do they set themselves up for more speech errors in the future? Questionnaire data from 334 respondents suggest that they do. Respondents whose names shared initial or final sounds with a sibling’s reported that their parents accidentally called them by the sibling’s name more often than those without such name overlap. Having a sibling of the same gender, similar appearance, or similar age was also associated with more frequent name substitutions. Almost all other name substitutions by parents involved other family members and over 5% of respondents reported a parent substituting the name of a pet, which suggests a strong role for social and situational cues in retrieving personal names for direct address. To the extent that retrieval cues are shared with other people or animals, other names become available and may substitute for the intended name, particularly when names sound similar.

## Introduction

### Parents Accidentally Substitute Similar Sounding Sibling Names

We don’t choose our parents, we don’t choose our first language, but we often get to pick the names of our children. So, we cannot help it if our parents are embarrassing, if our language has some inconvenient vocabulary quirks, but in many cultures we determine whether our offspring end up with similar sounding first names such as *Jason* and *Justin or Marie* and *Mary*.

Personal names serve to differentiate individuals but also to socially categorize them [1]. In about a third of the world’s cultures, members of a family traditionally share a surname. By tradition in some cultures, siblings receive given or first names that share a syllable or orthographic character to mark that they belong to the same family or a particular generation within a family (e.g., [2], [3]). Even in the absence of such a tradition, some parents make a point of giving their children similar sounding first names. An extreme example comes from the American reality television Duggar family which includes 19 children with names starting with the letter *J* and pronounced/dz/(as does the father’s name; http://duggarfamily.com/). Giving siblings phonologically similar names may increase the sense of family unity. Although much work has considered how names affect self-identity, social categorization, and social interactions [1], little is known about the consequences of personal name choice on speaking [4]. This study begins to fill the gap.

How quickly and accurately a word can be generated depends on its semantic, syntactic, associative, and phonological relationships with other words in a speaker’s vocabulary (e.g., [5], [6]; for review, see [7]). When word substitutions occur, they tend to involve words that belong to the same semantic category as the intended word even if the words sound nothing alike (e.g., labeling a lion *tiger* [8]). When naming objects, labels for visually similar members of the same category are more likely to intrude than less visually similar ones are. When a member of the same category as the intended word also overlaps in sound (e.g., *pear* for *peach*; *directory* for *dictionary*), it is more likely to substitute for the intended word than predicted if word representations were selected based on meaning independent of sound. (Note that words that share morphemes such as *bi* in *bicycle* and *biweekly* are considered as a different class of error.) This *mixed semantic and phonological error effect* has been observed in spontaneous speech error corpora [9]-[11], as well as speeded object-naming experiments with unimpaired participants and aphasic speakers (e.g., [12]-[15] but see [16]).

A mixed semantic and phonological error effect has also been observed when speakers retrieve personal names, but defining semantic similarity among people can be complicated. Celebrities are often used to study the retrieval of personal names. They are categorized primarily by occupation and nationality [17], [18]. So, calling one performer by the name of another performer is a common error and considered a semantically related name substitution. For example, Brédart and Valentine [18] examined the substitutions made when participants were asked to produce the last names of celebrities as quickly and accurately as possible. Participants made more mixed semantically and phonologically related name substitutions (i.e., [actress Marilyn] *Monroe* for [mime Marcel] *Marceau*) than expected if semantic substitutions were only phonologically related to intended names by chance. Mixed substitution errors occur in spontaneous speech and may attract considerable attention, particularly when they involve political leaders. For example, the name [Barack] *Obama* was frequently substituted for the name of another similar sounding, contemporary leader’s name, *Osama* [bin Laden] (for discussion see [19]). In addition, an interview and diary study found significantly more cases of mistaken identity and misnaming for people who had similar sounding names [20].

For general vocabulary and the names of strangers, the existence of words that overlap in meaning and sound is a property of the language that is outside of an individual speaker’s control. The following study investigated whether giving siblings similar names increases the tendency for their parents to accidentally substitute one sibling’s name for another. The current study differs from earlier studies of personal name substitutions in focusing on parents addressing their children by the wrong name and the phonological properties of the names. Furthermore, by querying the misnamed individuals about errors rather than querying speakers, we could compare the properties and circumstances of people who do not recall any name substitution errors with those who do.

So, the primary goal was to test whether parents who gave their children similar sounding names were more likely to address one sibling by the other sibling’s name. A second goal of the study was to explore influences on personal name substitutions in daily life. In particular, we relate predictors of name substitutions to research in social psychology and psycholinguistics.

## Method

### Ethics Statement

Documentation of written consent was waived because the survey was administered via the Internet. Participation posed no risk. Agreeing to participate in the survey was a prerequisite for viewing it. Responses were preserved digitally with other data. The study (H07122) was approved by the Institutional Review Board at the Georgia Institute of Technology.

### Participants

Only data from respondents aged 18 or older, who provided complete information about at least one sibling were considered in the analyses reported here. Data from five identical twins were excluded under the assumption that their parents’ name substitutions might be mistakes in person identification rather than name selection errors. Analyses are based on data from a total of 334 respondents. Of these, 231 adults (*M* age = 37.3 years, *SD* = 12.6) were recruited from mailing lists concerned with language research and received no compensation, and 103 were undergraduates at the Georgia Institute of Technology (*M* age = 19.7, *SD* = 1.4), who received extra credit in an introductory psychology course.

### Materials and Data Treatment

The questionnaire was administered via Surveymonkey.com. Questions asked about “parent(s)/guardian(s)”. We asked for legal first names and commonly used nicknames for the respondent and up to four siblings (or other children who lived in the same household for 2 or more years), starting with the one closest in age to the respondent. For simplicity, we will use *parent* and *sibling* to refer to these relationships. Most respondents (56%) had only one sibling. Analyses were limited to the sibling closest in age to the respondent, because such siblings seemed likely to provide the most opportunities for substitution errors. Questions asked for current age in years and gender (male or female) of the respondent and sibling, the number of years spent living with a parent and the sibling, the number of years elapsed since living with a parent, 7-point ratings of similarity in appearance and personality between respondent and sibling, and a frequency rating of a parent making an accidental sibling name substitution within the last 12 months (*no opportunity, never, once, a couple of times, once a month, once a week or more*) and across the lifetime of the respondent (*never, rarely, occasionally, often, very often*).

After questions about siblings, respondents were asked about any other name a parent called them by mistake, whether they were called by the name of a parent, names that other people had called them by mistake, and name substitutions that they had made. Many of the later questions were optional, open-ended, or ambiguously worded, making them unsuitable for quantitative analysis. This report is limited to data related to name substitutions made by parents.

Responses of *often* (11.7%) and *very often* (4.8%) for the lifetime frequency of sibling name substitutions were collapsed into one category labeled, *often*. The ratings of similarity in appearance and personality were reduced to 4 levels to eliminate categories with few observations. These ratings were treated as ordinal predictors.

We also asked which name (legal or nickname) a parent was most likely to use if substituting a sibling’s name. First names and nicknames were phonemically transcribed. Overlap in initial and final phonemes was coded for the name that the parent most commonly used for the respondent and the name of the sibling reported as typically used in substitutions or in general. We also coded whether the respondent’s and sibling’s names had the same number of syllables and stress pattern.

## Results and Discussion

Analyses were based on data for the sibling that was closest to the respondent in age (either older or younger). Forty-four percent of respondents reported that a parent accidentally called them by the name of the sibling at least once within the last year. However, 13% of respondents had no opportunity for a parent to do so. So, analyses reported here focused on whether respondents reported that a parent *never* (21%), *rarely* (39%), *occasionally* (24%), or *often* (16%) called them by the sibling’s name across the respondents’ lifetimes.

Data were analyzed with cumulative odds ordinal regression models that estimate the influence of predictors on the odds of being at or above a category across cumulative splits of the outcome variable [21]. For example, one split would test the odds for *never* versus at least *rarely* being called by the sibling’s name. Alpha was set to 0.10 for Wald χ^2^ values for retaining single predictors in stepwise backwards elimination. Then plausible 2-way interaction terms for retained predictors were added with stepwise forward inclusion. The assumption of parallel or proportional odds was supported by comparing the best fitting predictors from the ordinal regression models with the same predictors in a multinomial regression model (*AICc* = 813 vs. 840). Table 1 displays the parameters for the best fitting ordinal regression model. In other words, only significant and marginally significant predictors are listed in Table1.

**Table 1:** Parameters for the best fitting cumulative odds ordinal regression model for lifetime frequency of sibling name substitutions by a parent.

Predictor	*b (log-odds)*	*SE(b)*	Odds ratio	Wald χ^2^	*df*	*p*
Same initial phoneme	0.309	0.161	1.362	3.68	1	0.055
Same final phoneme	0.302	0.149	1.353	4.13	1	0.042
Same final phoneme x Same gender	0.319	0.149	1.376	4.60	1	0.032
Same gender	0.626	0.154	1.870	16.62	1	<0.001
Age difference in years	−0.112	0.042	0.894	7.11	1	0.008
Same gender x Age difference	−0.098	0.042	0.906	5.56	1	0.018
Similarity in appearance (4–5≥)	0.215	0.301	1.240	11.47	3	0.009
Similarity in appearance (3–4)	0.496	0.317	1.642	−	−	−
Similarity in appearance (≤2–3)	0.132	0.290	1.141	−	−	−
First born	−0.245	0.106	0.782	5.33	1	0.021
Years since lived with parent	0.020	0.008	1.020	5.74	1	0.017
Intercept (*never* vs. *rarely* or more)	−2.358	0.354				
Intercept (*rarely* or less vs. *occasionally* or more)	−0.133	0.332				
Intercept (*occasionally* or less vs. *often*)	1.332	0.339				

*N* = 334. Log Likelihood χ2 (11) = 109.09, *p*<.0001. *R* ^2^_L_ = 0.122, generalized *R* ^2^ = 0.299.

### Did Respondents’ Parents give Siblings Similar Sounding Names?

The initial sound in the first name or nickname of 12% of respondents was the same as the initial phoneme in their sibling’s name. This overlap was slightly but significantly greater than expected if the initial sounds of the siblings’ names were randomly paired. This was tested with Kappa’s measure of agreement in which the initial sounds of a respondent’s name and a sibling’s name were considered as categorizations that could match or mismatch (*K* = 0.059, *p*<.0001). The final phoneme in the name of 16% of respondents was the same as the final phoneme in their sibling’s name. We considered whether this final overlap might be attributed to the diminutive suffix/i/(as in *Bobby*) used in many nicknames. Although/i/was the most common final sound across names, the final sounds schwa and/n/were shared more often by siblings, as in *John* and *Catherine*. This pattern suggesting that the overlap was often solely phonological rather than morphological. The frequency of name-final phoneme overlap did not differ significantly from chance, *K* = 0.020, *ns*. Thus, sibling names in this dataset were consistent with the idea that some parents intentionally select sibling names that share initial sounds, but overlap in final sounds may arise by chance due to many common names having the same final sounds.

### Was Phonological Similarity between Sibling Names Associated with More Frequent Name Substitutions?

**Figure 1:**
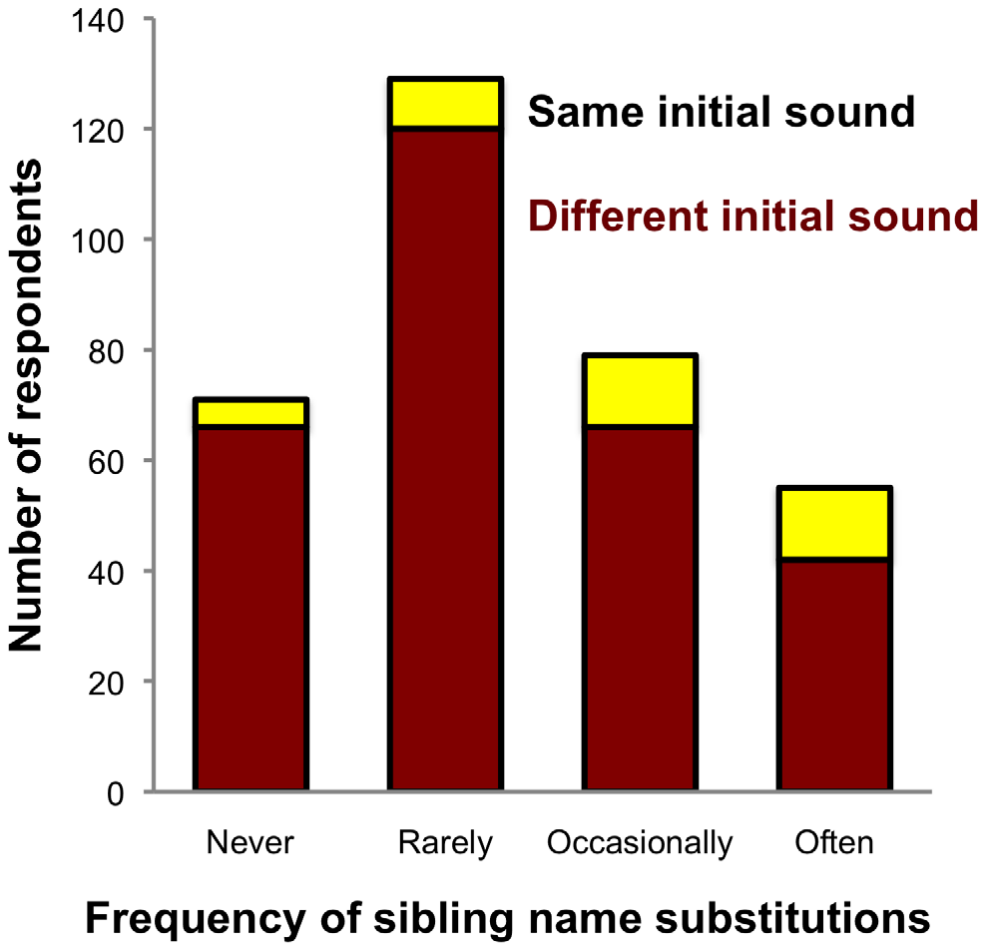
Frequency distribution of respondents with and without the same initial phoneme in their names as their closest sibling in age across the reported lifetime frequency of name substitutions made by their parents.

As shown in Figure 1 and Table 1 (*Same initial phoneme*), names with shared initial phonemes were associated with higher name-substitution frequencies than were names with different initial phonemes. Only 7% of respondents who reported that a parent never called them by their sibling’s name had names that shared initial sounds with their sibling’s. In contrast, 24% of the respondents who reported that a parent often called them by a sibling’s name shared initial sounds. The effect of sharing an initial sound did not interact with other factors such as sharing the same last sound, having the same gender, age, or physical similarity. Sharing the final sound of the sibling’s name predicted higher substitution frequencies, particularly for siblings of the same gender, which was reflected in a significant interaction between the variables for gender and final sound (see *Same final phoneme* and *Same final phoneme x Same gender* in Table 1). Only 4% of people who reported never being called by their sibling’s name shared both gender and their name’s final sound with their sibling, whereas 15% of people reporting substitutions often did. The effect of sharing a final sound did not interact with other factors such as age or physical similarity.

Although word substitutions often involve words with the same number of syllables and stress patterns [10], these variables did not predict the frequency of sibling name substitutions. We did not test whether a higher number of shared sounds initially or finally further increased substitutions because greater phonological overlap was uncommon. Altogether the results support the hypothesis that both intentional and unintentional overlap in the sounds of siblings’ names makes parents more likely to call one sibling by the other’s name.

### Did Sibling Similarity Increase Substitutions?

Previous research suggests that accidental name substitutions and mistakes about people’s identities tend to involve people with the same gender, same race, and similar age [3], [20]. Extrapolating from that work, we expected name substitutions to increase with similarity between siblings in gender, age, and rated similarity in appearance. The analyses bore out this prediction (see Same *gender, Age difference in years, Same gender x Age difference*, and *Similarity in appearance* in Table 1). The influence of gender was particularly strong. Siblings with the same gender comprised 15% of respondents reporting never being called by their sibling’s name and 78% of those reporting that they were often called by their sibling’s name. The effect of age difference was amplified when siblings had the same gender, which was reflected in an interaction between the variables for same gender and age difference. Aside from potentially contributing to physical similarity, gender and age are social variables that speakers use to select terms of address (e.g., *mister* vs. *ma’am* vs. *miss*). So, the importance of gender and age as social categories (e.g., [22], [23]) may have influenced substitutions rather than or in addition to their potential contribution to visual similarity.

Although it seemed plausible that substitutions would increase with ratings of similarity in personality, there was no significant association. This may be due in part to the crude rating used, *How similar this sibling is to you on a scale from 1 to 7 in personality (interests, habits)?* and the relative weakness of personality as a grouping variable.

### How did Birth Order and History of Name use Affect Name Substitutions?

From a parent’s perspective, siblings are members of the category [MY CHILDREN]. First-born children are the sole members of this category at least briefly, which may give their names an advantage due to earlier entry into the parent’s daily vocabulary and possibly greater cumulative frequency of use compared to the names of later children. These properties of personal names are similar to the variables *age of acquisition* (i.e., the age at which a word is learned) and *word frequency* (i.e., how often a word is used) that influence word substitution errors when labeling objects [24]. Thus we hypothesized that first-born children might be less likely to suffer sibling name substitutions. Indeed, first-born children reported less frequent name substitutions than later-born children did (see *First born* in Table 1), comprising 63% of the *never* category but only 42% of *often*. However, as a study relying on memory for events, the observed associations between variables may be influenced by memory biases. For example, younger siblings may take greater umbrage at being called by an elder sibling’s name than the reverse, resulting in the difference in reported substitution rates.

We asked participants how long ago they lived with their parents, based on the suspicion that longer intervals would be associated with fewer opportunities for name substitutions, poorer memory for substitutions, or both. Unexpectedly, we saw the reverse; more years away from parents predicted higher name substitution frequencies (see *Years since lived with parent* in Table 1). There are many plausible potential explanations for this pattern. Respondents’ ages were highly correlated with their number of years living away from their parents, *r*(333) = 0.96, *p*<0.0001. The current age of the parent was not solicited, but we suspect it was likely highly correlated with both respondent age and time spent living apart. So, one possibility is that sibling name substitutions increase with a speaker’s (i.e., parent’s) age as name retrieval failures do [25]. The same association between time living apart from the parent appeared in analyses of substitutions frequencies during the past 12 months (*b* = 0.054, *SE* = 0.012, Wald χ^2^ = 18.87, *p*<.0001), which suggests that the effect was not due to time-inflated memories of substitutions that occurred primarily while living at home. The pattern is also consistent with a memory bias favoring recall of substitution errors made by older parents, perhaps due to concerns about dementia.

We considered it possible that the number of years spent living with the sibling might be correlated with these variables and influence the opportunities for errors to occur and therefore predict substitution frequency. Counter to this expectation, the number of years living with the sibling was not significantly correlated with the number of years spent living with a parent and only weakly correlated with the respondent’s age, *r*(333) = 0.11, *p*<.05. In addition, there was no significant effect of years living with the sibling in an additional model substituting years living with parents with years living with sibling (*b* = –0.0035, *SE* = 0.031, Wald χ^2^ = 0.01, *p*>.9).

### Did Having a Nickname Make Substitutions More or Less Likely?

Most theories of word production posit that words with similar meanings compete with one another for selection. For example, all else being equal, it takes longer to produce the name of an object with synonymous names (e.g., *sofa*/*couch*) than one with a single dominant name (e.g., *apple*; see [26], [27]). Along the same lines, a person’s nickname might compete with their legal name, increasing the time needed to generate a name and increasing its vulnerability to speech errors.

We hypothesized that respondents who reported that their parents did not commonly call them by their legal names might report more sibling name substitutions. There was no significant effect of having a nickname or not, but this may have been due to mixing together nicknames of differing difficulty. For example, abbreviated nicknames such as *Griff* for *Griffin* are easier to learn and faster to retrieve than unrelated nicknames such as *Toby* for *Junius* (Davison & Griffin, unpublished data).

Similarly we predicted that parents with more children might suffer more competition when trying to select the name of a single child. However, there was no significant effect of number of siblings.

### Were Substitution Rates Similar for Males and Females?

We did not expect a difference in substitution frequencies based on gender, but did expect people to ask about it. There was no significant effect of the respondent’s gender.

### What Other Name Substitution Errors did Parents Make?

Respondents were also asked if they recalled a parent accidentally called them, “by the name of someone (or something) other than a sibling.” In particular, we wondered whether parents other than those of the second author addressed their children with pets’ names by mistake. In case respondents recalled multiple non-sibling name substitutions, they were asked to report the one that was used most often. One hundred twenty one respondents reported being called by a non-sibling’s name. When asked about the relationship between the parent and the owner of the substituted name, 78.5% of those respondent’s mentioned family relationships such as being called by the name of the parent’s spouse, sibling, or grandchild (see Table 2). Surprisingly, 20 respondents reported that a parent accidentally called them by the name of a family pet (primarily dogs, a few cats, and one horse). One person described the relationship between the parent and name-owner only as “friend” and another reported that there was no relationship between the speaker and the owner of the name.

**Table 2:** Accidental non-sibling name substitutions categorized by relationship (*N* = 121).

Relationship	*N*	%
Parent’s spouse and/or respondent’s parent	41	33.9
Parent’s sibling and/or respondent’s aunt or uncle	38	31.4
Pet belonging to parent, respondent, or family	20	16.5
Other kin	16	13.2
Friend	1	0.8
No relationship	1	0.8
Unclear or uninformative response	4	3.3

In asking about the gender of the owner of the name, we provided the options of *male*, *female*, and *not human*. The majority of the human owners of names had the same gender as the respondent (88%), echoing the strong influence of gender in sibling name substitutions. Because non-sibling names were inconsistently reported, their phonological overlap with respondents’ names was not coded. It is worth noting however that reported names of pets included several that would be unusual for a human, such as *Fluffy*.

Nominal logistic regression models were used to test whether other information we collected might distinguish between respondents who reported that their parents accidentally called them by the name of a pet rather than another human. We hypothesized that pets’ and humans’ names might be in complimentary distribution with pets’ names being used more when siblings or other family members were unavailable or unlikely substitutes for some reason. The results did not support this the hypothesis. The majority (65%) of the respondents who were called by the name of a pet had an elder sibling, whereas only 34.7% who were called a non-sibling human’s name had an elder sibling; a significant difference (*b* = –0.558, *SE* = 0.263, Wald *χ^2^* = 4.509, *p* = 0.034). Furthermore, the respondents who were called a pet’s name tended to report higher lifetime frequencies of being called by the name of their closest sibling in age (Mdn = 2, *occasionally*) compared to respondents who were called by another human’s name (Mdn = 1, *rarely*; *b* = –0.529, *SE* = 0.247, Wald *χ^2^* = 4.582, *p* = 0.032). None of the six other variables tested were robust predictors of pet-name substitutions (the number of years since the respondent lived with a parent, whether the respondent had a nickname, number of siblings, age difference with the closest sibling in age, same gender as closest sibling, and respondent’s gender).

So, respondents with an elder sibling or who were frequently called by another sibling’s name were more likely to report that a parent called them by a pet’s name rather than a human’s name. This might be due to frequent sibling name substitutions making pet-substitution errors more salient and memorable to respondents. It also may be that parents who substitute pets’ names for their children’s names are particularly prone to make name substitutions in general.

## Conclusion

As expected, the results suggest that when a parent attempts to retrieve the name of his or her child, the process is influenced by many of the same variables as when a speaker labels an object. In particular, we were interested in whether phonological similarity between the siblings’ names increased the frequency of accidental name substitutions. Indeed, when a respondent’s name shared the initial or final sound with the name of a sibling, parents substituted the sibling’s name more often. Combined with existing research on mixed semantic and phonological effects in word substitutions (e.g., [5], [18], [28]), this suggests that parents increase their likelihood of calling their children by each other’s names when they have given them names that sound alike.

Other predictors of name substitutions also appeared analogous to the results of word production experiments. For example, rated similarity in appearance increased the frequency of parents substituting one child’s name for another. Most sibling name substitutions and substitutions of other family members’ names occurred when name-bearers had the same gender. Gender and age are often reflected in appearance so the effects of these variables may reflect physical similarity, but their importance for social identity and terms of address may amplify their role in personal name retrieval in a way that does not have an obvious analog in labeling objects. Younger siblings were called by a sibling’s name or a pet’s name more often than first born children were. This advantage for first born children may be due to their names having higher frequency of use, analogous to word frequency effects in other speech errors [24] and in accord with the pervasive impact of experience on behavior in general. Family membership, like category membership for objects, appeared to be a strong influence on word substitutions with only two respondents reporting that a parent called them by the name of a person from outside the family, broadly construed.

The most intriguing and unexpected result for the authors was the number of respondents who reported that parents called them by the name of a pet. Anecdotally, Fiske et al. [20] also noted some households in which children were mistakenly called by the names of dogs. It is tempting to attribute such mistakes to the animals’ status as family members and child-substitutes (see [29]). However, it seems unlikely that parents would make such errors so readily if they were labeling family members in photographs. Overlap in discourse context and communicative intentions seem likely to promote the substitutions. Specifically, utterances directed at pets typically include their names in order to get their attention and summon them [30]. In open-ended questions, some respondents mentioned pet name substitutions occurring when they were being summoned. We hypothesize that overlap in the discourse function of name use promotes the substitution errors. In addition, speech directed to human family members and pets has many characteristics in common and that differ from speech to others, even close friends. For example, interactions with family members and pets often take place in the home and sometimes under exceptionally emotional circumstances. The unexpected prevalence of pet-name substitutions suggests that situational or social similarity may influence name retrieval powerfully enough to yield name substitutions despite obvious differences in the species of the name-bearers.
